# Comment to: “Propofol and survival: an updated meta-analysis of randomized clinical trials”: authors’ reply

**DOI:** 10.1186/s13054-023-04528-0

**Published:** 2023-06-15

**Authors:** Yuki Kotani, Alessandro Pruna, Todd C. Lee, Dominik Roth, Giovanni Landoni

**Affiliations:** 1 Department of Anesthesia and Intensive Care, IRCCS San Raffaele Scientific Institute, Via Olgettina 60, 20132 Milan, Italy; 2 School of Medicine, Vita-Salute San Raffaele University, Via Olgettina 58, 20132 Milan, Italy; 3 Department of Intensive Care Medicine, Kameda Medical Center, 929 Higashi-Cho, Kamogawa, Chiba 296-8602 Japan; 4 Division of Infectious Diseases, Department of Medicine, McGill University, Montreal, QC Canada; 5 Department of Emergency Medicine, Medical University of Vienna, Währinger Gürtel 18-20, 1090 Vienna, Austria

Our meta-analysis of RCTs in critically ill and perioperative patients [1] suggested propofol is associated with a relative mortality increase of 10% highlighting the potential for harm acknowledged since 2001 [2] but which has been relatively forgotten. We have reported multiple subgroup and sensitivity analyses which all supported the magnitude (approximately 10%) and direction (against propofol) of the survival effect.

Systematic reviews usually aim to cover all available evidence. Meta-analyses offer the opportunity to demonstrate smaller effects which may not be identified in small- or middle-sized randomized trials. For instance, the mortality benefit of low molecular weight heparins in severe COVID-19 patients was not demonstrable in the initial trials but has been shown in meta-analysis [3]. The issue of variation in follow-up time is frequently encountered in clinical trials. Outside of individual patient meta-analyses which might use time-to-event and regression analyses, mortality is consequently usually dichotomized, assuming the relative effect between intervention and control are constant across the entire observation time [4]. Mortality will almost inevitably increase over time in both groups as Gutierrez et al. pointed out.

In the context of pooling studies with different follow-up times, this leaves two options: (1) pooling only studies with the same mortality time-point, leading to lower sample sizes and precision and therefore reducing one of the main strengths of meta-analyses or (2) pooling all studies irrespective of follow-up time. Specifically, in the field of critical care and perioperative medicine, it has been shown that different mortality time points did not influence pooled point estimates. Limiting analyses to only one time point would, however, decrease precision and generalizability of the findings [5].

We are co-authors on the Shehabi et al. and Schaefer et al. studies and are aware that young patients requiring high-doses of propofol in the ICU or in perioperative settings have a low mortality. As these were not randomized comparisons, we think this is proxy of being healthy and not an indication that propofol was the safest sedation agent.

As proposed by Gutierrez et al., a network meta-analysis of thousands of trials and dozens of comparisons and settings would truly be a monumental feat! Nonetheless, given questions of propofol safety predate this century and millions are exposed annually, it might be better to apply those energies into generating the multicentered pragmatic randomized controlled trials required to truly advance the safety of practice.

## Data Availability

Further information on the original manuscript is available from the corresponding authors upon reasonable request.

## Abbreviation

ICU: Intensive care unit
